# Mars exploration on the move

**DOI:** 10.1093/nsr/nwaa181

**Published:** 2020-09-12

**Authors:** Mu-ming Poo

**Affiliations:** Director, CAS Center for Excellence in Brain Science and Intelligence Technology, China; Executive Editor-in-Chief of NSR

While the world is still struggling to overcome the COVID-19 pandemic, a new round of Mars exploration proceeded as planned, catching the July–August 2020 window of Earth–Mars close proximity that occurs once every 26 months. The excitement began with United Arab Emirates’ launch of the *Hope* orbiter on 19 July, followed by China's launch of the *Tianwen 1* (天问一号) explorer on 23 July, and NASA’s launch of the *Perseverance* rover on 30 July. All these probes will reach the vicinity of Mars seven months later. The successful launch of Emirates’ *Hope* orbiter and China's *Tianwen 1* explorer into the Earth-to-Mars Hohmann transfer orbit marks the beginning of Mars exploration by these two countries, whereas the launch of NASA’s *Perseverance* rover is the continuation of a long series of Mars missions of the US that began in 1964.

The Emirates’ *Hope* orbiter was built through a collaboration between Mohammed bin Rashid Space Center in United Arab Emirates and three US universities, and it was launched at Tanegashima Island launch site using Japan's top-of-the-line H2A rocket. When it reaches Mars’ orbit, the probe will thoroughly investigate Mars’ atmosphere and weather events such as dust storms in the lower atmosphere. It will answer the questions of how and why the weather varies in different regions of Mars, and why Martian atmosphere is losing hydrogen and oxygen into space.

The *Tianwen 1* Mars explorer, named after the title of a poem by the ancient Chinese poet Qu Yuan, ‘Questions to Heaven’, was launched at Wenchang launch site in Hainan Providence using China's most powerful ‘Long March 5’ (长征五号) carrier rocket. This is an ambitious ‘three steps in one’ attempt to combine circulating, landing and roving of Mars into one mission. The *Tianwen 1* explorer includes an orbiter and a land explorer comprising an entry module and a rover, and it will perform exploration both in the orbit and on land, either independently or in coordination. The orbiter and rover carry a variety of cameras, detectors, spectrometers and particle analyzers to investigate the planet's ionosphere, magnetic field, geological structure, soil and mineral compositions, underground distribution of water/ice, and cosmic ray particles.

*Perseverance* is NASA’s fifth Mars rover, launched from Cape Canaveral Air Force Station in Florida using a United Launch Alliance Atlas V rocket. It is designed to search for astrobiological evidence of ancient microbial life on Mars. When it lands at Jezero Crater, the rover will gather rock and soil samples for future return to Earth and will investigate the planet's climate and geology and pave the way for future human exploration of Mars. Of particular interest is the light-weight *Ingenuity Mars Helicopter* that will be deployed by the rover for testing the feasibility of powered, controlled flight in the Martian atmosphere, whose density is only 1% that of the Earth. If *Ingenuity's* flight is successful, future robotic flying vehicles could offer unique low-altitude views of Mars not provided by orbiters and rovers.

Space exploration presents an ideal platform for inter-national collaboration. The best example is the International Space Station (ISS), participated in by 16 countries including Russia, US, Japan, Canada and Brazil and 11 member countries of the European Space Agency. Many scientific projects were carried out in the ISS, and the most notable one is the Alpha Magnetic Spectrometer (AMS) project led by Samuel C.C. Ting. On board ISS since 2011, AMS has collected a large amount of data on cosmic ray particles that may shed light on the nature of dark matter and antimatter. Seven Chinese research institutions have participated in the AMS international team, and the Chinese Academy of Sciences (CAS) Institute of Electrical Engineering, the Institute of High-Energy Physics, and China Academy of Launch Vehicle Technology have built a key component of ASS—a large permanent magnet, the largest example of such equipment ever shipped into space.

Understanding the mystery of the universe has been a dream of humanity ever since the dawn of civilization. Perhaps more than any other science, astronomy captures the imagination of all curious minds. Mars exploration signifies humanity's quest in the scientific frontier. It is often characterized in the media as a ‘space race’, linked to the technological competition among superpowers, but it should really be a race in the Olympian spirit. We will all get to the finishing line and share the excitement and achievement together, as exemplified by the memorable scenes of multinational astronauts working together in the confined space of the ISS. Sometime in the distant future, a space station will appear on Mars as an extension of the harmonious global village, where people of all nations and races will work together to fulfill the dream of humankind.

